# Evaluation of bovine leukaemia virus infection control measures in Japanese Black beef cattle breeding farms: A 7‐year longitudinal study based on proviral load

**DOI:** 10.1002/vro2.70029

**Published:** 2026-02-22

**Authors:** Makoto Kajisa, Chikako Tani, Mineto Tani, Jiazhou Li, Satoshi Ito, Takenori Yamauchi, Hironobu Murakami, Hiromu Katamoto, Reiichiro Sato, Shingo Nakahata

**Affiliations:** ^1^ M&Y Animal Service Soo Japan; ^2^ Division of HTLV‐1/ATL Carcinogenesis and Therapeutics, Joint Research Center for Human Retrovirus Infection Kagoshima University Kagoshima Japan; ^3^ South Kyushu Livestock Veterinary Medicine Center, Joint Faculty of Veterinary Medicine Kagoshima University Soo Japan; ^4^ Department of Hygiene, Public Health and Preventive Medicine Showa University School of Medicine Tokyo Japan; ^5^ School of Veterinary Medicine Azabu University Sagamihiara Japan; ^6^ Department of Animal Pharmaceutical Sciences, School of Pharmaceutical Sciences Kyushu University of Medical Science Nobeoka Japan; ^7^ Graduate School of Medicine and Veterinary Medicine University of Miyazaki Miyazaki Japan

**Keywords:** barn layout, bovine leukaemia virus, Japanese Black beef breeding cattle, proviral load, real‐time polymerase chain reaction

## Abstract

**Background:**

Bovine leukaemia virus (BLV) infection remains a major problem for Japanese Black (Wagyu) cattle, and effective and sustainable control measures are essential to prevent its spread in breeding herds. This study aimed to monitor the BLV infection status of all cattle at a Japanese Black breeding farm in Kagoshima Prefecture, Japan, over 7 years.

**Methods:**

BLV serological testing was conducted at baseline in 2018 (Y0, study year 0), followed by real‐time polymerase chain reaction (PCR) testing in six survey years between 2019 and 2024 (Y1‒Y6; no testing in 2023). The infection status and proviral load (PVL) were assessed. Cattle were classified as calves (<10 months) or breeding cattle (≥10 months), reflecting standard Japanese production practices. After high‐PVL cattle were identified in Y1, new barns were constructed stepwise from Y2 onwards to physically separate BLV‐positive and BLV‐negative animals. In addition, strategic culling of BLV‐positive cattle, BLV screening of introduced cattle, and early separation of calves from their dams were implemented.

**Results:**

At Y0, BLV seroprevalence among breeding cattle was 78.6%. At Y1, PCR detected BLV in 28.0% of calves and 74.1% of breeding cattle. By Y6, BLV prevalence had significantly decreased to 39.2% in breeding cattle compared with Y1, while it was 21.6% in calves, with no significant reduction observed.

**Conclusions:**

Early identification of high‐PVL cattle, physical separation of BLV‐positive and BLV‐negative cattle, and culling of BLV‐positive cattle was effective in reducing BLV prevalence in a Wagyu breeding herd. These findings propose a feasible, farm‐based approach for managing endemic BLV under commercial conditions.

## INTRODUCTION

Enzootic bovine leukosis (EBL) is a chronic viral disease caused by the bovine leukaemia virus (BLV), which belongs to the family *Retroviridae*. Bovine leukaemia virus is genetically related to human T‐cell leukaemia virus type 1 (HTLV‐1)^1^ and is primarily transmitted horizontally through infected blood. Its vectors include haematophagous insects, and its vehicles include contaminated needles.^2^, ^3^ Vertical transmission via the placenta, birth canal, colostrum and milk has also been documented.^4^, ^5^, ^6^, ^7^ Most infected cattle remain asymptomatic. However, some develop EBL and exhibit clinical signs such as enlargement of the peripheral lymph nodes, emaciation, reduced milk yield, diarrhoea and lethargy.^8^, ^9^

Enzootic bovine leukosis is endemic in many countries worldwide and significantly affects livestock production.^8^, ^10^ It was designated a notifiable infectious disease under the Act on Domestic Animal Infectious Diseases Control in Japan in 1998. The Ministry of Agriculture, Forestry and Fisheries issued national hygiene guidelines for controlling the spread and introduction of BLV on farms in 2015.^11^ Bovine leukaemia virus was introduced to Japan via imported dairy cattle. Ichijo suggested that dairy cows imported from the United States were the source of infection in eastern Hokkaido in the 1960s.^12^ A sero‐epidemiological survey conducted in 2007 revealed an overall BLV prevalence of 28.6% based on the results of tests for 5420 cattle (3966 dairy cows, 797 beef breeding cows and 657 beef fattening cattle) from 209 farms across seven prefectures in Japan. The prevalence was higher for the dairy cows (34.7%) than for the beef cattle (16.3% for the breeding cows and 7.9% for the fattening cattle).^13^ Furthermore, an epidemiological study conducted in 2013 reported prevalences of 40.9% and 28.7% for the dairy cows and beef cattle, respectively, indicating that BLV infection had become widespread during the 2000s.^14^ Ohno et al.^15^ reported that high proviral loads (PVLs) were detected in 73.3% of the 1417 BLV‐positive cattle sampled from farms in a nationwide study conducted between 2012 and 2014 in Japan. Cases of EBL in young cattle (aged <3 years) have been reported in recent years, highlighting the increasing importance of early infection control measures.^16^

Japanese Black (Wagyu) cattle play a central role in Japan's beef industry, especially in the southwestern warm regions. Kagoshima Prefecture is one of the leading production areas, with approximately 123,100 Japanese Black breeding cows. They account for nearly 20% of the national total (Japan Ministry of Agriculture, Forestry and Fisheries, Livestock Statistics 2024).^17^ Bovine leukaemia virus research has primarily focused on dairy cattle.^5^, ^18^ However, studies involving Japanese Black cattle have been gradually increasing.^19^, ^20^ Notwithstanding, data on BLV infection dynamics and the effectiveness of control strategies for Japanese Black breeding herds are limited, especially at the farm level.

Reports on the epidemiology of BLV in beef cattle and its effects on productivity have been increasing overseas. A survey in Kansas, United States, found high antibody prevalence and many high‐PVL animals in beef herds.^21^ There are also reports indicating that BLV infection does not clearly impair reproductive performance,^22^ as well as studies suggesting that infected cattle may have reduced longevity.^23^ These findings highlight the importance of implementing BLV control measures in beef cattle as well.

This study aimed to monitor the BLV infection status of all cattle on a Japanese Black breeding farm in Kagoshima for 7 years after implementing practical control measures, including the segregation of BLV‐positive and BLV‐negative animals. These results will provide valuable insights into effective on‐farm strategies for BLV control in Japanese Black beef cattle management. They will also provide insights into a practical framework that can be adapted for use with beef cattle herds in other endemic regions worldwide to support BLV control and eventual eradication.

## METHODS

### Study site and farm description

This study was conducted at Farm H, a family‐operated Japanese Black (Wagyu) beef cattle breeding farm located in a livestock‐intensive area of Kagoshima Prefecture, Japan. The farm specialised in cow–calf operations. All the calves were conceived via artificial insemination and raised on‐site. They were housed with their dams for approximately 3 months at the beginning of the study and were primarily nursed by their mothers during this period. However, they may have suckled other lactating cows because of the loose barn system.^24^

Most of the female calves were sold at approximately 10 months of age, as were the male calves. However, some were retained on the farm as replacement breeding animals. The farm also introduced breeding stock, including calves and pregnant cows, from local livestock markets to support genetic improvement. The calves that tested positive for BLV by polymerase chain reaction (PCR) were preferentially sold after the study commenced. In the breeding herd, culling was performed when BLV‐positive cattle showed poor health or had high PVL levels. However, depending on pedigree value, age or economic considerations, selective culling was not implemented in some BLV‐positive cattle.

**FIGURE 1:**
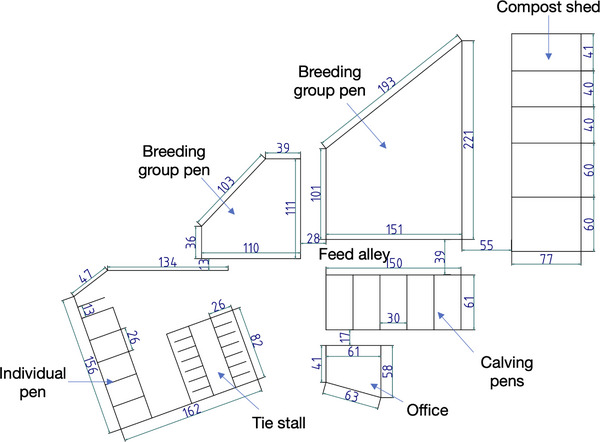
Farm layout at the beginning of the study. The old barn was initially constructed as a tie stall. It was subsequently expanded to include calving pens, a compost shed, a breeding group pen and individual pens. The roofs of all sections, except for the compost shed, are continuous and overlapping. Ventilation is natural and facilitated by fans. The dimensions in the figure are in units of 0.1 m.

**FIGURE 2:**
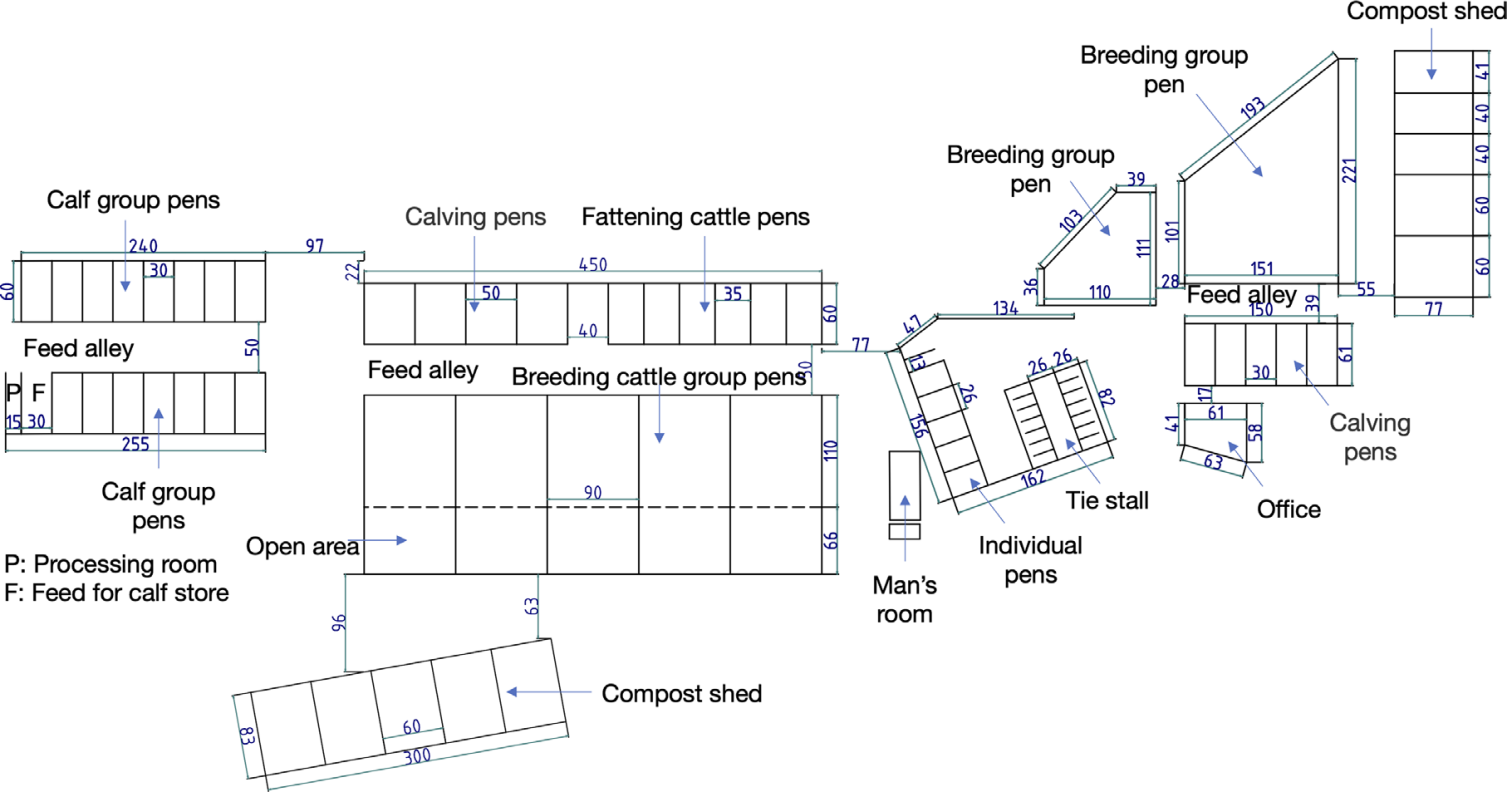
Farm layout 3 years after the beginning of the study. The new barn includes facilities for adult cattle (breeding group pens, calving pens, fattening cattle pens and a compost shed). A separate calf barn was constructed 1 year later. The calves are artificially fed for 3 months and reared for an additional 10 months. They are shipped as fattening cattle, except for the breeding heifers. The adult and calf barns are structurally independent. The cattle in the breeding group have access to an outdoor area. Ventilation is natural and facilitated by fans. The dimensions in the figure are provided in units of 0.1 m.

The facilities of the farm initially included designated spaces for calves, growing young cattle, breeding cattle and periparturient cattle, all these were located within a single barn structure (Figure 1). A new barn for breeding cattle was constructed in the second year of the study (Y2), and a dedicated calf barn was constructed in the third year (Y3) (Figure 2). The calf management system was modified beginning in Y3. The calves were allowed to stay with their dams for the first week postpartum to receive colostrum and maternal milk. They were subsequently separated and raised in the calf barn on milk replacer until they were 3 months old.

The farm was managed by six individuals: four family members (the owner couple and two relatives) and two part‐time staff. The farm owner had basic knowledge of BLV, but diagnostic testing had not been conducted before this study. The attending veterinarian applied strict disinfection protocols during rectal examinations, parturition assistance and treatment procedures, and implemented appropriate medical hygiene measures, including the use of disposable needles and gloves and appropriate management of equipment used in dehorning. In addition, no system was in place in the study area to test for BLV, including testing of newly introduced cattle, during the study period. One case of EBL was confirmed on the farm in the year preceding the study. This prompted the owner to recognise the need for herd‐wide infection monitoring and led to the implementation of this research.

### Animal sampling

Bovine leukaemia virus testing was conducted annually from 2018 to 2024. The tests were performed on 11 May 2018, 13 April 2019, 12 September 2020, 25 July 2021, 14 May 2022 and 9 June 2024. We used the farm's existing age‐based management groups (calves <10 months; breeding cattle ≥10 months, including replacement heifers and adult cows) for the analysis. The average ages and standard deviations for each year are provided in Table 1. A total of 502 animals (187 calves and 315 breeding cattle) were examined over the 7 testing years.

**TABLE 1:** Summary of bovine leukaemia virus infection status in farm H (2018–2024).

Year	Detection method	Group	Negative (–)	Positive (+)	Total	Prevalence (%)	Mean age (months)
2018 (Y0)	Ab test	Calves	11	20	31	64.5^a^	7.5 ± 2.9/3.6 ± 2.3
		Breeding	9	33	42	78.6	48.2 ± 33.6/79.7 ± 37.9
2019 (Y1)	LTR (PCR)	Calves	18	7	25	28.0	6.0 ± 2.8/4.8 ± 1.4
		Breeding	14	40	54	74.1^b^	45.7 ± 45.6/75.4 ± 40.0
2020 (Y2)	LTR (PCR)	Calves	29	4	33	12.1	4.2 ± 2.2/5.1 ± 3.4
		Breeding	19	38	57	66.7^b^	43.9 ± 40.1/88.1 ± 38.1
2021 (Y3)	pol (PCR)	Calves	28	11	39	28.2	4.9 ± 2.7/5.1 ± 3.7
		Breeding	26	37	63	58.7^b^	47.5 ± 44.6/84.4 ± 41.0
2022 (Y4)	pol (PCR)	Calves	35	5	40	12.5	4.6 ± 3.2/6.5 ± 2.4
		Breeding	32	34	66	51.5^b^	46.0 ± 43.9/75.8 ± 38.4
2024 (Y6)	pol (PCR)	Calves	40	11	51	21.6	3.7 ± 2.7/5.1 ± 3.1
		Breeding	45	29	74	39.2^a^, ^b^	44.5 ± 23.6/73.4 ± 34.4
2019–2024	Mixed	Calves	150	38	188	20.2	4.5 ± 2.8/5.2 ± 2.9
		Breeding	136	178	314	56.7	45.5 ± 37.5/79.8 ± 38.6

*Note*: Calves—under 10 months; breeding—10 months or older. Mean ages are denoted as mean ± standard deviation for negative and positive animals. Mixed: multiple polymerase chain reaction (PCR) methods (long terminal repeat [LTR] and/or pol) used during 2019–2024.

^a^ Infection status in 2018 based on antibody test; maternal antibodies may affect results.

^b^ Significant year‐to‐year difference in breeding group infection prevalence (*p* < 0.001).

^c^ Significant pairwise differences in breeding group infection prevalence (2019 vs. 2024, 2020 vs. 2024).

The turnover rate, as an indicator of the degree of herd structural change each year, was calculated as follows:

$$\mathrm{Turnover}\;\mathrm{rate}=\frac{\mathrm{total}\;\mathrm{number}\;\mathrm{of}\;\mathrm{introduced},\;\mathrm{sold}\;\;\mathrm{and}\;\mathrm{culled}\;\mathrm{animals}\;\mathrm{during}\;\mathrm{the}\;\mathrm{calendar}\;\mathrm{year}\;\left(1\;\mathrm{January}-31\;\mathrm{December}\right)}{\mathrm{number}\;\mathrm{of}\;\mathrm{animals}\;\mathrm{kept}\;\mathrm{on}\;\mathrm{the}\;\mathrm{inspection}\;\mathrm{date}\;\mathrm{in}\;\mathrm{that}\;\mathrm{year}}\;\times 100$$

In this study, records managed on a calendar‐year basis by farmers and livestock hygiene service centres were used to determine the numbers of introduced, sold and culled animals; therefore, the numerator represents the total number for the calendar year. In contrast, the denominator—the number of animals kept—was based on the herd size recorded on the inspection date conducted each year (the inspection month varied by year). Although the time frames of the numerator and denominator do not completely coincide, the same method was consistently applied across all years; thus, the validity of comparisons among herds and across years was considered to be maintained.

### Virological testing

Blood samples were collected from the jugular vein of each animal. Those collected in 2018 (Y0) were drawn into plain tubes containing separation reagents. The serum was separated on the same day, and the serum samples were analysed for BLV antibodies via enzyme‐linked immunosorbent assay (ELISA). The serum samples were stored at ‒30°C until analysis. The blood samples collected in EDTA tubes from 2019 (Y1) to 2024 (Y6) were stored at 4°C and later subjected to real‐time PCR for detection and quantification of proviral DNA.

The PVLs were determined using the long terminal repeat (LTR)‐based method with the CoCoMo‐BLV detection kit (RIKEN) in 2019 (Y1) and 2020 (Y2).^25^, ^26^ They were determined using the pol gene‐targeted PCR method in 2021 (Y3), and the results were expressed as copies per 10 ng of DNA.^27^ The pol method was used exclusively in 2022 (Y4) and 2024 (Y6), and the PVL values were reported as copies per 100,000 cells.^28^

The three different PVL quantification methods used during the study period were due to the outsourcing of testing to different laboratories (Department of Hygiene, Public Health and Preventive Medicine; Showa University School of Medicine, School of Veterinary Medicine, Azabu University; Graduate School of Medicine and Veterinary Medicine, University of Miyazaki), each of which employed its own validated protocols. Proviral load testing was performed at affiliated laboratories in some cases as part of ongoing research collaborations. Nevertheless, all PVL data were categorised as very low, low, moderate or high based on the criteria reported by Nishimori et al.^28^ to ensure consistent interpretation. Therefore, differences in testing methods did not affect the classification of BLV transmission risk or the assessment of BLV prevalence.

The thresholds for the LTR method were as follows: less than 1000 copies/100,000 cells (very low), 1000 to less than 5000 (low), 5000 to less than 20,000 (moderate) and 20,000 or more (high). The thresholds for the pol method were as follows: less than 600 copies/100,000 cells (very low), 600 to less than 3000 (low), 3000 to less than 12,000 (moderate) and 12,000 or more (high).

### Statistical analysis

The 2 × *n* contingency tables for the calf and adult groups were constructed based on their BLV‐positive and BLV‐negative statuses for each year from 2019 to 2024. The Fisher–Freeman–Halton test, which is applicable to *r* × *c* contingency tables, was used to evaluate the overall differences in BLV prevalence across years. The pairwise differences across the years were assessed using Fisher's exact test applied to the 2 × 2 contingency tables. Exact tests were used for all comparisons because the sample sizes and expected frequencies varied by year. The null hypothesis for each pairwise comparison was that the BLV prevalence was the same for the 2 years. The alternative hypothesis was that the prevalence for at least one of the years was different. The *p*‐values were adjusted using the Holm method to control for the inflation of type I error due to multiple testing, and statistical significance was set at an adjusted *p*‐value of less than 0.05.

A supplementary analysis was conducted to assess the influence of differences in age structure between the groups on BLV prevalence. The mean ages (in months) of the BLV‐positive and BLV‐negative cattle were determined for each year and group. The data were compared using Welch's *t*‐test. The differences in mean age with infection status across the years were assessed using one‐way analysis of variance and Tukey's post hoc test. Statistical analyses were performed using R software (version 4.3.3).

## RESULTS

### Annual changes in BLV prevalence after the introduction of BLV control measures

The annual prevalence of BLV was evaluated in the calf group (under 10 months of age) and the breeding cattle group (10 months of age or older, including replacement heifers and adult cows) from 2018 (Y0) to 2024 (Y6). In Y0, BLV infection was assessed using an ELISA‐based antibody test; from Y1 onwards, BLV status was determined solely by PCR testing. Annual BLV positivity rates and PVL data are summarised in Tables 1, 2, 3.

**TABLE 2:** Summary of proviral load (PVL) in bovine leukaemia virus (BLV)‐positive cattle (2019–2024).

Year	Group	BLV‐positive headcount	Mean PVL (copies)	Mean age (months)
2019 (Y1)	Calves	7	20,020 ± 21,022	4.9 ± 1.5
	Breeders	40	28,959 ± 22,637	76.5 ± 40.6
2020 (Y2)	Calves	4	10,504 ± 14,854	5.2 ± 3.4
	Breeders	38	34,532 ± 34,172	90.5 ± 38.5
2022 (Y4)	Calves	5	59,032 ± 32,048	5.8 ± 2.3
	Breeders	34	58,765 ± 61,802	75.4 ± 38.4
2024 (Y6)	Calves	11	23,314 ± 32,963	5.1 ± 3.1
	Breeders	29	30,026 ± 41,640	74.5 ± 34.9

*Note*: PVL values are expressed as mean ± SD. Units vary by assay method.

**TABLE 3:** High proviral load (PVL) in calves with bovine leukaemia virus who are younger than 5 months.

Year	Age (months)	PVL (copies/10^5^ cells)	PCR method
2019	3.6	11,450.1	LTR‐based PCR
2019	4.6	19,129.4	LTR‐based PCR
2022	3.5	61,325.9	pol‐based PCR
2024	4.2	9202.6	pol‐based PCR
2024	4.6	16,952.9	pol‐based PCR

*Note*: The calves listed in this table had medium to high transmission risk based on their elevated PVL.

Abbreviations: LTR, long terminal repeat; PCR, polymerase chain reaction.

Screening by BLV antibody testing in 2018 (Y0) revealed a high BLV positivity rate of 78.6% in the breeding cattle group, indicating that BLV infection had already spread extensively within the farm. Therefore, in 2019 (Y1), to accurately assess the BLV infection status on this farm, BLV PVL testing using real‐time PCR was conducted. As a result, 28.0% of calves and 74.1% of breeding cattle were determined to be BLV positive (Table 1). Among the BLV‐positive breeding cattle, approximately 60% exhibited a high PVL (Table 2) and were considered the main sources of infection contributing to viral spread. In 2020 (Y2), a new barn for breeding cattle was constructed, enabling the separate housing of BLV‐positive and BLV‐negative cattle based on PVL (Figure 2). At the same time, culling was initiated, particularly targeting BLV‐positive cattle with high PVL, and this resulted in a reduction in BLV prevalence in the breeding cattle group (Table 1).

In 2021 (Y3), a calf‐dedicated barn was constructed, and a management system in which calves were separated from their dams immediately after birth and housed according to the BLV infection status of the dams was established. Calves born to BLV‐negative dams were assigned to the BLV‐negative group without testing, whereas calves born to BLV‐positive dams underwent PCR testing early after birth and were housed as the BLV‐positive group. In addition, all introduced cattle were tested for BLV and assigned to either the BLV‐positive or BLV‐negative group. These measures further strengthened the segregation management system and reduced the risk of new infections.

In 2022 (Y4), a policy was introduced whereby PCR testing was performed at shipment at 10 months of age, and only BLV‐negative animals were selected as replacement cattle. Following the introduction of these control strategies, BLV prevalence in the breeding cattle group gradually decreased throughout the study period, reaching 39.2% in 2024 (Y6) (Table 1). In contrast, BLV prevalence in calves fluctuated from year to year, and no consistent decreasing trend was observed.

From 2019 (Y1) to 2024 (Y6), a total of 38 BLV‐positive calves were identified, of which 42.1% were determined to have been infected before 5 months of age (Table 1). Among these calves, four exhibited extremely high PVL and were classified as medium‐ to high‐risk animals (Table 3). These results indicate that some calves are infected with BLV immediately after birth and maintain high viral loads at an early stage, supporting the importance of early monitoring and segregation management for calves born to BLV‐positive dams.

### Herd expansion and replacement rate

From 2019 (Y1) to 2024 (Y6), the size of the breeding cattle herd gradually increased due to planned herd expansion on the farm. Data on cattle introduction, culling and replacement during this period are shown in Table 4. Although the annual replacement rate varied, it was overall low, with an average of 10.6%, reflecting limited animal movement within the herd. The highest replacement rate was observed in Y4 (20.3%), while the lowest was observed in Y1 (5.6%).

**TABLE 4:** Annual introductions, removals, herd size and turnover rates in calf and breeding groups (2019–2024).

Year	Group	Introduced (head)	Culled/sold (head)	Total herd size (head)	Breeding group turnover rate (%)	Breeding group net increase (head)
2019 (Y1)	Calf	4	31	25	–	–
	Breeding	1	3	54	5.6	12
2020 (Y2)	Calf	2	39	33	–	–
	Breeding	0	4	57	7.0	3
2021 (Y3)	Calf	10	40	38	–	–
	Breeding	5	6	64	9.4	7
2022 (Y4)	Calf	8	40	40	–	–
	Breeding	4	13	66	20.3	2
2024 (Y6)	Calf	1	55	51	–	–
	Breeding	0	7	74	10.6	8
Total/mean	Calf	25	205	187	–	–
	Breeding	10	33	315	10.6 (mean over years)	32

### Statistical analysis

In the calf group, no significant differences in BLV prevalence were observed between years. In contrast, significant differences were observed in the breeding cattle group, with BLV prevalence being significantly lower in later years than in the initial years (Table 1). In a supplementary analysis comparing age distributions between BLV‐positive and BLV‐negative cattle, no significant differences were observed in the calf group in any year. In contrast, in the adult cattle group, BLV‐positive cattle were consistently older than BLV‐negative cattle. Significant age differences were detected in 2019 (+30 months, *p*_adj = 0.043), 2020 (+44 months, *p*_adj = 0.0013), 2021 (+37 months, *p*_adj = 0.0046), 2022 (+30 months, *p*_adj = 0.001) and 2024 (+29 months, *p*_adj = 0.0013). However, ANOVA analysis showed no consistent temporal trend in these age differences.

## DISCUSSION

This study demonstrated that, between 2019 and 2024, BLV prevalence could be effectively reduced in a Japanese Black breeding farm by combining regular PCR testing with the following management interventions that considered the risk of BLV transmission. The main measures included whole‐herd testing of BLV PVLs, physical separation of BLV‐positive and BLV‐negative cattle, prioritised culling of BLV‐positive cattle with high PVLs, BLV screening of introduced cattle, separation of calves from their dams immediately after birth, and a policy of selecting only BLV‐negative animals as replacement cattle.

Before the initiation of this study, the farm adopted a policy of ‘managing without BLV testing’, and the BLV infection status of the herd was unclear. When BLV antibody testing was conducted for all cattle, widespread BLV infection was suggested. Subsequently, the introduction of annual PCR testing enabled accurate identification of BLV‐infected cattle as well as individuals at high risk of BLV transmission. Together with the expansion of the breeding cattle barn, this allowed the implementation of separate housing for BLV‐positive and BLV‐negative cattle and selective replacement with BLV‐negative heifers. In particular, identifying and culling cattle with high PVLs has been reported to be a highly effective strategy for reducing BLV prevalence over several years in dairy herds.^29^ In addition, the construction of a dedicated calf barn enabled BLV testing based on the BLV infection status of the dams and segregation management according to the test results. These measures demonstrate that BLV control is feasible in Japanese Black breeding farms by combining testing and management strategies, even without the complete culling of all infected cattle.^30^

However, in this study, BLV prevalence in calves did not decrease significantly. This is likely attributable to the continued vertical transmission from high‐PVL dams that remained within the breeding herd.^6^, ^7^ Among calves diagnosed as BLV‐positive before 5 months of age, several exhibited medium to high PVLs (Table 3), suggesting the involvement of dam‐to‐calf transmission (transplacental, intrauterine or via the birth canal).^4^, ^6^, ^7^ In addition, annual variation in BLV prevalence among calves may have been influenced by which cows were used for breeding in a given year and by the timing of BLV testing in calves, as BLV‐positive calves were preferentially sold; however, vertical transmission is considered to have been the dominant contributing factor. These findings emphasise the importance of early calf separation and continued culling of high‐PVL adult cattle for reducing vertical transmission.

The most effective approach for BLV control is the culling of all infected cattle^30^, ^31^; however, in Japanese Black breeding farms, where the economic value of individual animals is high, a simple ‘test‐and‐cull’ strategy is not realistic. This study demonstrated that meaningful reductions in BLV prevalence can be achieved while maintaining economic viability by combining regular testing, strategic culling of high‐PVL individuals, selective replacement and physical separation of BLV‐positive and BLV‐negative cattle.

## LIMITATIONS

Several limitations of this study should be acknowledged. The precise relative contributions of vertical and horizontal transmission in calves could not be clearly evaluated because detailed cohabitation data and the infection status of all contact animals were not available. In addition, herd expansion and within‐year fluctuations in herd size may also have influenced BLV prevalence estimates. Replacement rates were calculated on a calendar‐year basis and did not necessarily correspond to herd size at the time of testing. Moreover, in year 5, PVL testing was not performed for all animals due to practical constraints; consequently, data from that year were excluded from the analysis, resulting in a gap in the chronological sequence of the study. Furthermore, different primers were used for quantitative PCR in different years, which may have affected PVL quantification to some extent. Other potential risk factors, such as cohabitation with BLV‐infected cattle, housing density and selective shipment of BLV‐positive calves,^30^, ^31^ were also not fully evaluated in this study.

## CONCLUSIONS

The findings of this study strongly indicate that, in Japanese Black breeding farms where BLV is widespread, substantial reductions in BLV prevalence can be achieved by combining continuous PVL monitoring with practical management interventions, including segregation of BLV‐positive and BLV‐negative cattle, prioritised culling of high‐PVL individuals, screening of introduced cattle and early separation of dams and calves. Although controlling early‐life infection in calves remains challenging,^3^, ^32^ the long‐term reduction in BLV prevalence in the breeding herd supports the effectiveness and feasibility of this approach. This strategy represents a practical and economically viable model for Japanese Black breeding farms and may provide valuable insights for the development of BLV control strategies at regional or national level.

## AUTHOR CONTRIBUTIONS

Makoto Kajisa, Chikako Tani and Shingo Nakahata contributed to the conception and design of the study. Makoto Kajisa, Mineto Tani, Takenori Yamauchi, Hironobu Murakami and Reiichiro Sato acquired the data. Makoto Kajisa, Chikako Tani, Mineto Tani, Jiazhou Li, Satoshi Ito, Takenori Yamauchi, Hironobu Murakami, Hiromu Katamoto, Reiichiro Sato and Shingo Nakahata analysed and interpreted the data. Makoto Kajisa, Chikako Tani, Satoshi Ito and Shingo Nakahata drafted the manuscript. Satoshi Ito performed the statistical analysis. All the authors reviewed and edited the manuscript and approved the final version for submission.

## CONFLICTS OF INTEREST

The authors declare they have no conflicts of interest.

## FUNDING INFORMATION

The authors received no specific funding for this work.

## ETHICS STATEMENT

This study was approved by the Animal Experiment Review Board of the University of Miyazaki (approval number: 2024‐034), Japan. The standards for the care and maintenance of industrial animals in Japan were followed.

## Data Availability

The data that support the findings of this study are available from the corresponding author upon reasonable request.
